# Swallowing function management in patients with disorders of consciousness: a scoping review

**DOI:** 10.3389/fnins.2025.1595393

**Published:** 2025-04-16

**Authors:** Hua Long, Bixia Lu, Qiao Tan, Dongmei Dai, Fengfei Zeng

**Affiliations:** ^1^Department of Clinical Nursing, Second Xiangya Hospital, Central South University, Changsha, China; ^2^Department of Neurosurgery, Second Xiangya Hospital, Central South University, Changsha, China; ^3^Xiangya Boai Rehabilitation Hospital, Changsha, China

**Keywords:** disorders of consciousness, swallowing disorder, scoping review, assessment, intervention

## Abstract

**Objective:**

Patients with disorders of consciousness often have concurrent swallowing difficulties, although the assessment methods, interventions, and their effectiveness have not been systematically described. This study aims to conduct a comprehensive review of assessment methods and rehabilitation interventions for swallowing function in patients with disorders of consciousness.

**Methods:**

This scoping review was performed and reported according to the Preferred Reporting Items for Systematic Reviews and Meta-Analyses extension for Scoping Reviews (PRISMA-ScR) guideline. Studies that describe a screening method to examine swallowing function or assess a kind of swallowing management intervention among individuals with disorders of consciousness (DoC) were included. Following Arksey and O’Malley’s five-stage framework, databases including CNKI, WangFan, VIP, SinoMed, PubMed, Web of Science, Embase, ClinicalTrials.gov, Cochrane, Scopus, and Medline were systematically searched, from the inception of each database to June 2024. Relevant studies were extracted and analyzed. The main review question is “What has been studied about swallowing function management in patients with DoC?”.

**Results:**

Assessment methods for swallowing function in patients with disorders of consciousness primarily included clinical swallowing assessments, scale-based evaluations, and instrument-based assessments. Rehabilitation interventions for swallowing function encompassed sensory stimulation, K-point stimulation, functional oral intake therapy, oral intermittent tube feeding, neuromuscular electrical stimulation, and acupuncture therapy. Most of the included studies did not explicitly specify the timing of swallowing assessment and intervention.

**Conclusion:**

A standardized approach for evaluating and intervening in swallowing function among patients with disorders of consciousness is notably lacking. Selecting appropriate swallowing assessment tools and devising evidence-based management plans tailored to assessment results could improve the swallowing function and patient outcomes. More high-quality designing research that compares the assessment accuracy of different evaluation methods, as well as develops personalized interventions are imperative.

**Systematic review registration:**

https://doi.org/10.17605/OSF.IO/SURBY.

## 1 Introduction

Disorders of consciousness (DoC) are states of impaired consciousness caused by various severe brain injuries, including coma, vegetative state (VS) or unresponsive wakefulness syndrome (UWS), and minimally conscious state (MCS) (China Association of Rehabilitation of Disabled Persons, China Association of Rehabilitation Medicine, China Rehabilitation Research Center, 2023). It is reported that in developed countries, the incidence of DoC is 0.2 to 6.1 cases per 100,000 people; while in developing countries such as China, approximately 70,000 to 150,000 new DoC cases emerge annually, with total medical costs exceeding 30 to 50 billion yuan (Zhao, 2020). Research has indicated that patients with disorders of consciousness exhibit disruptions in the central nervous system, leading to dysfunctions in protective reflexes, such as disorders in swallowing (Yang et al., 2017). Swallowing Disorder refers to the difficulty in eating due to the inability to safely and effectively transport food from the mouth to the stomach to obtain sufficient nutrition and hydration (Chinese Rehabilitation Medicine Association Swallowing Disorder Rehabilitation Professional Committee, 2023). Studies show that over 99% of DoC patients have swallowing disorders (Mélotte et al., 2021). These disruptions often result in impaired oral, pharyngeal, and esophageal phases of swallowing, significantly increasing the risk of aspiration pneumonia, malnutrition, and dehydration (Wang et al., 2019; Raciti et al., 2022). Recently, experts have recommended the early implementation of swallowing management in patients with DoC to maintain or enhance their swallowing function (Zhang and Ling, 2023), given the fact that the initial recovery of swallowing function serve as the indicator of conscious recovery in DoC patients. Therefore, effective management of swallowing function is a cornerstone of care for the population with DoC.

Accurate evaluation of swallowing function is essential for tailoring safe and effective swallowing management strategies. The swallowing function of DoC patients is dynamically changing (Mélotte et al., 2021), increasing the complexity and heterogeneity of the evaluation. A wide variety of assessment tools and protocols are currently employed, ranging from bedside evaluations (e.g., Kubota Drinking Test) (Wang et al., 2019) to advanced instrumental methods (e.g., videofluoroscopy) (Peters et al., 2021). The aforementioned tools are applicable to different patient groups, such as those who can actively cooperate or those requiring specific examinations. The heterogeneity in assessment practices and the lack of standardized guidelines create inconsistencies in clinical decision-making and hinder optimal patient care.

Similarly, swallowing rehabilitation interventions in patients with DoC remain an evolving area of study. Techniques such as sensory stimulation, and neuromodulation have shown promise, but their efficacy and applicability are often limited by inconsistent findings and methodological variability in the literature. Without a clear understanding of the evidence supporting these approaches, clinicians face challenges in selecting appropriate and effective interventions.

Considering a lack of evidence-based research that systematically summarizes the swallowing function management strategies among DoC patients (Zhang and Ling, 2023), this study seeks to address these critical gaps by comprehensively examining the assessment methods and rehabilitation interventions for swallowing function in patients with DoC (Mandaville et al., 2014). By synthesizing the current state of evidence, this review aims to lay the groundwork for developing standardized, evidence-based rehabilitation programs for improving swallowing management in the vulnerable population with DoC.

## 2 Methods

This scoping review was performed and reported according to the Preferred Reporting Items for Systematic Reviews and Meta-Analyses extension for Scoping Reviews (PRISMA-ScR) guidelines. Literature review follows Arksey and O’Malley’s five-stage framework (Arksey and O’Malley, 2005) for scoping reviews: (1) identifying the research question according to the patient, concept and context framework, (2) identifying relevant studies, (3) study selection, (4) charting the data and (5) collating, summarizing and reporting the results. Protocol of the present scoping review has been registered.^1^

### 2.1 Identifying the research question

The blueprint for this review is “What has been studied about swallowing function management in patients with DoC?” The more detailed research questions were as follows: Q1. What are the current assessment methods used to evaluate swallowing function in patients with DoC, and what is the optimal timing for using them? Q2. What rehabilitation interventions are available for improving swallowing function in patients with DoC, and how effective are these interventions?

### 2.2 Identifying relevant studies

The patient, concept and context framework was followed to identify studies:

1) Participants: This review considered studies of individuals with DoC, including the status of coma, VS, UWS, and MCS.
2) Concepts: Studies that describe a screening method to examine swallowing function or assess a kind of swallowing management intervention among individuals with DoC.
3) Context: Studies conducted in hospitals, rehabilitation centers, nursing home, clinical outpatient settings etc. were considered in this review.

**Figure 1:**
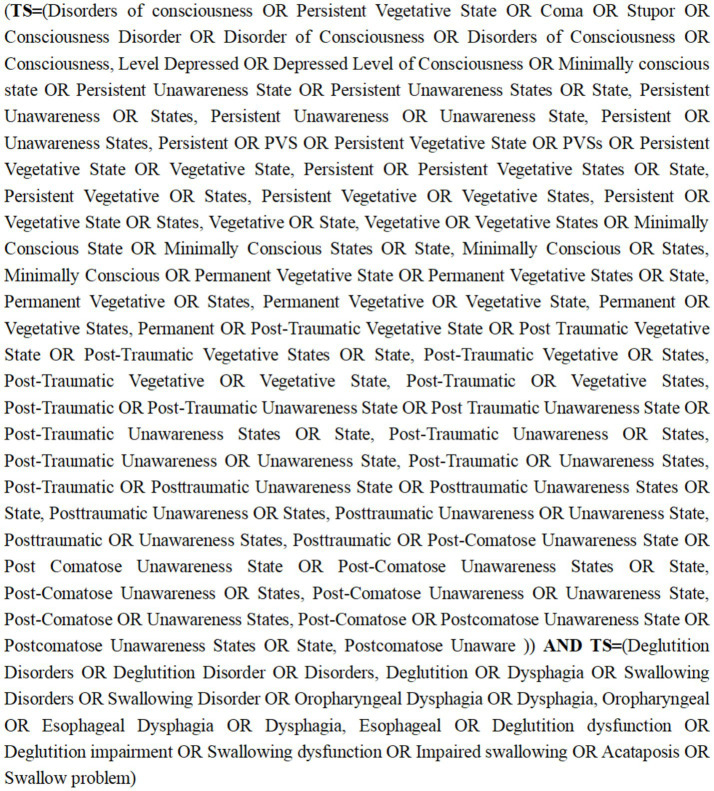
Search strategy for Web of Science.

A systematic search of 11 electronic databases PubMed, Web of Science, Embase, Clinical Trials, Cochrane, Scopus, Medline, CNKI, WangFan, VIP, and SinoMed was conducted from the inception of the databases to June 2024. Each of these platforms offers a unique coverage of literature, ensuring a comprehensive collection of relevant literature. For each database, we developed an adequate research string that combined the terms “Disorders of Consciousness,” “Persistent Vegetative State,” “Coma,” “Consciousness Disorder,” “Deglutition Disorders” and “Swallow Problem.” We defined our search strategy with the collaboration of health sciences research librarians. The detailed search strategies for all included databases are displayed in Supplementary material, and are exemplified by the use of Web of Science below (Figure 1).

### 2.3 Study selection and eligibility criteria

To be included, the identified records have to (1) include subjects who are patients with DoC (Glasgow Coma Scale score 3 to 8) and are at least of 18 years old; (2) involve swallowing function assessment, and swallowing function rehabilitation interventions in patients with DoC; (3) be published in Chinese and English; (4) adopt a study design of randomized controlled trials, quasi-experimental studies, cross-sectional surveys, cohort studies, or case-control studies; and (5) we show preference for the most recent and highest quality literature of the same type.

Exclusion criteria were: (1) studies conducted on organic diseases of the oropharynx, psychological factors, oral ulcers or dryness, medications affecting saliva secretion or mental state, frailty or muscle atrophy due to old age, diseases related to esophageal swallowing disorders; (2) studies including patients with cancer; (3) studies including patients with tracheal intubation; (4) literature types are guidelines, reviews, conference abstracts, or proposals; (5) full text is unavailable.

After importing all retrieved literature into Note Express to remove duplicates, two evidence-trained researchers independently screened them based on inclusion and exclusion criteria. Initially, a preliminary screening was conducted by reading titles and abstracts, followed by a full-text review for further screening. In cases of disagreement on study inclusion, a third reviewer was consulted to reach a consensus using a principle of minority yielding to the majority through voting. Specifically, in addition to the title, abstracts and full-text of articles identified from the database search, we also reviewed study titles citing the accepted articles for possible inclusion as well as their respective reference lists. The risk of bias or the quality of the studies included was not formally assessed in our review because scoping reviews, unlike systematic reviews, aim at answering broad questions and critical appraising of the evidence would fall beyond the aims of a scoping review.

### 2.4 Data extraction

Two independent researchers extracted data from the studies that were ultimately included, based on the research questions (i.e., Q1: the current evaluation methods used to evaluate swallowing function in patients with DoC, and Q2: rehabilitation interventions available for improving swallowing function in patients with DoC). Therefore, the extracted data for studies that address the evaluation of swallowing function included author, year of publication, type of study design, study location, study subjects, sample size, assessment tool, time, and evaluator. For studies that could be used for swallowing function intervention integration, extracted data included author, publication year, type of study design, study location, subjects and sample size, intervention duration and method, intervention implementer, and outcome assessment method related to swallowing function. Discrepancies in the process of data extraction were discussed and resolved in consultation with a third researcher.

### 2.5 Synthesis

The results were synthesized using a thematic analysis with consideration to the objectives that guided the scoping review.

## 3 Results

### 3.1 Results of literature search

**Figure 2:**
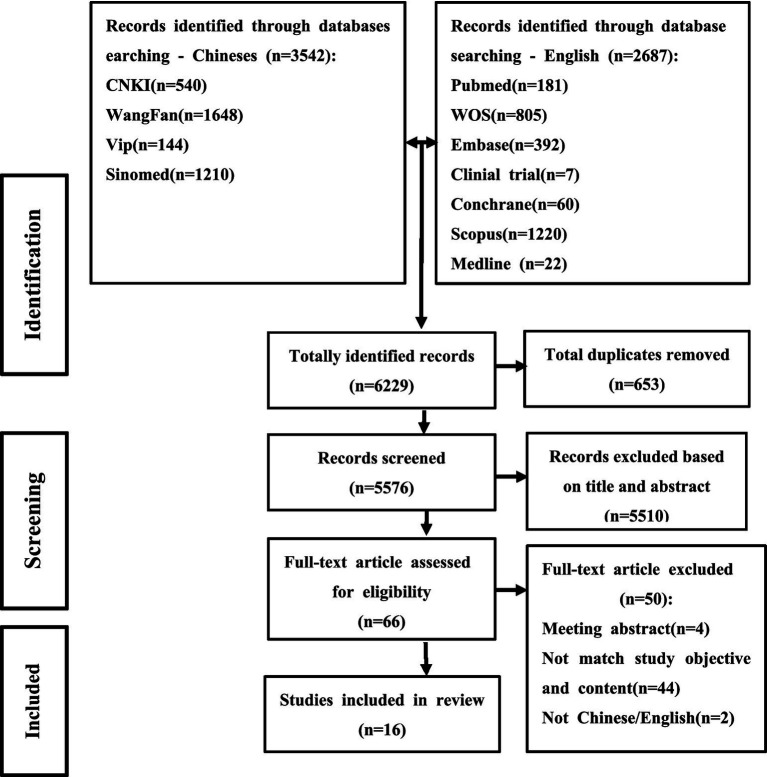
Flow chart of study selection process.

A total of 6,229 articles were initially retrieved, and after the removal of duplicates, 5,576 were screened for inclusion. From these, 5,510 records were excluded after reading the title and abstract, leaving 66 articles to be assessed for eligibility. After reading the full-text, 44 studies were excluded because their research objectives and content were not aligned with the scope of this review; four studies were excluded as they were conference abstracts; and two studies were excluded due to being written in languages other than Chinese or English. Finally, 16 articles were included in this scoping review. The literature screening process is depicted in Figure 2.

### 3.2 Basic characteristics of included literature

Among the 16 included articles, nine are descriptive studies and seven are interventional studies. The descriptive studies primarily focus on the assessment of swallowing function in patients with disorders of consciousness (DoC), and include three from France, two from China, one from the USA, and one each from the USA, Belgium, Japan, and South Korea, published between 2014 and 2024, with sample sizes ranging from 11 to 92. In contrast, the seven interventional studies address the intervention of swallowing function management in DoC patients. These studies include five from China, one from Denmark (a pilot study), and one from Switzerland, published between 2016 and 2023, with sample sizes ranging from 10 to 63. The study subjects encompass patients with hemorrhagic stroke, ischemic stroke, and traumatic brain injury. The basic characteristics of the included studies are detailed in Tables 1, 2.

**Table 1:** The basic characteristics of included studies on swallowing function evaluation (*n* = 9).

Author	Year of publication	Research design	Research setting	Sample/cases	DoC type	Disease diagnosis	Evaluate time	Swallowing evaluation		
								Assessment method	Reference tools	Assessor
Mélotte et al. (2021)	2021	Retrospective design	Belgium	92	UWS MCS	Stroke/trauma	—	Clinical evaluation	FEES	Clinicians
Wang et al. (2019)	2019	Self-controlled study	China	19	UWS MCS	Stroke/trauma	—	KDT	—	Neurologist
Peters et al. (2021)	2014	Retrospective design	America	60	Coma	Trauma	>6 weeks after brain trauma	Clinical evaluation	VFSS	Clinicians
Bremare et al. (2016)	2016	Prospective design	France	11	Coma	Trauma	61 days after brain trauma	Clinical evaluation	FEES	Physical therapist
Zhang N. et al. (2021)	2019	Prospective design	China	60	UWS	—	—	Staining	KDT	Nurse
Prum et al. (2023)	2023	Retrospective design	France	33	UWS MCS	Stroke/trauma	1 month after admission	FOIS	FEES VFSS	Clinicians
Moriyama et al. (2023)	2023	Retrospective design	Japan	53	Coma	Stroke	—	FOIS	VFSS	Physical therapist
Jang et al. (2023)	2023	Retrospective design	Korea	51	UWS MCS	Stroke	>6 weeks after stroke	PAS	VFSS	Physical therapist
Herr et al. (2024)	2024	Prospective design	France/Belgium	14	UWS MCS	Stroke/trauma	—	SWADOC	SSA	Clinicians

DoC, disorders of consciousness; UWS, unresponsive wakefulness syndrome; MCS, minimally conscious state; VFSS, Video Fluoroscopy Swallowing Study; FEES, Fiberoptic Endoscopic Evaluation of Swallowing; KDT, Kubota Drinking Test; FOIS, Functional Oral Intake Scale; PAS, Penetration Aspiration Score; SWADOC, Swallowing Assessment in Disorders of Consciousness; SSA, Standardized Swallowing Assessment; —, not be described.

**Table 2:** The basic characteristics of included studies on swallowing function intervention (*n* = 7).

Author	Year of publication	Research design	Research setting	DoC types	Disease diagnosis	Sample/cases	Intervention method				Swallowing function outcome
							Implementer	Intervention content		Time	
								Control group	Experimental group		
Wu et al. (2016)	2016	Quasi-experiment	China	Coma	Stroke	93	Nurse	4	1	—	The swallowing frequency
Zhang et al. (2017)	2017	Experiment study	China	MCS	Stroke/trauma	30	Physical therapist	1 + 2	1 + 2 + 3	Four weeks	KDT
Jakobsen et al. (2019)	2019	Experiment study	Denmark	Coma	Trauma	19	Physical therapist	—	7	Three weeks	PAS FOIS SEMG
Zhang C. H. et al. (2021)	2021	Quasi-experiment	China	Coma	Stroke/trauma	60	Nurse /patient’s family	1	1 + 6	Four weeks	MMASA
Zhang et al. (2023)	2023	Quasi-experiment	China	MCS	Stroke/trauma	60	Physical therapist /nurse	1 + 2 + 3	1 + 2 + 3	Four weeks	KDT
Theytaz et al. (2023)	2023	Quasi-experiment	Swiss	UWS MCS	Stroke/trauma	28	Physical therapist	8	9	Eighteen months	SEMG
Wang et al. (2023)	2023	Quasi-experiment	China	Coma	—	63	Physical therapist	1	1 + 2 1 + 2 + 3	Four weeks	KDT SSA

DoC, disorders of consciousness; MCS, minimally conscious state; UWS, unresponsive wakefulness syndrome; KDT, Kubota Drinking Test; PAS, Penetration Aspiration Score; FOIS, Functional Oral Intake Scale; MMASA, Modified Mann Assessment of Swallowing Ability; SEMG, Surface Electromyography; SSA, Standardized Swallowing Assessment; 1, sensory stimulation therapy; 2, neuromuscular electrical stimulation therapy; 3, K-point stimulation; 4, oromotor exercises; 5, facial oral tract therapy; 6, intermittent ora-esophageal tube feeding; 7, nonverbal facilitation of swallowing; 8, palpation; 9, Nox-T3 device; —, not be described.

### 3.3 Swallowing function in patients with DoC

The studies included in the review investigated patients with various states of consciousness, including coma (seven studies), UWS (seven studies), and MCS (eight studies). The assessment tools for disorders of consciousness encompassed Glasgow Coma Scale, The Wessex Head Injury Matrix, and Coma Recovery Scale-Revised, etc. According to the swallowing function assessment tools employed and the consciousness state among patients, the prevalence of dysphagia varies widely, ranging from 30 to 100%. Notably, the prevalence of different types and locations of swallowing dysfunction varies. For example, Bremare et al. (2016) have reported the prevalence rates of oral and pharyngeal transport disorders, swallowing coordination problems, and swallowing regulation deficits, which are 77.8, 66.7, and 55.6%, respectively (Peters et al., 2021). Two additional studies investigated various factors influencing swallowing function in DoC patients (Prum et al., 2023; Moriyama et al., 2023). Peters et al. (2021) identified age, initial cognitive function grading, tracheotomy status, and vocalization ability as significant predictors of long-term swallowing difficulties in DoC patients. The area under the receiver operating characteristic curve for this predictive model was 0.81, indicating strong discrimination and high diagnostic accuracy. Moriyama et al. (2023) demonstrated that low skeletal muscle mass hinders swallowing function recovery in DoC patients, and also found that serum albumin levels upon admission correlate with swallowing recovery, with malnutrition identified as a risk factor for swallowing difficulties in DoC patients.

### 3.4 Swallowing function evaluation for patients with DoC

#### 3.4.1 Timing of swallowing function evaluation in DoC patients

The timing of swallowing evaluation plays a critical role in the management of dysphagia in patients with DoC. Through integration of the findings, we observed that only four studies addressed the timing of swallowing function assessment in DoC patients, with the initial time of swallowing function assessment mostly lies in 2–8 weeks after the state of consciousness changed, e.g., 6 weeks after swallowing problems appeared (Peters et al., 2021), 61 days post-onset (Bremare et al., 2016), within 8 weeks post-onset (Jang et al., 2023), and 1 month after admission (Prum et al., 2023). Almost half of studies failed to employ dynamic, regular assessments to evaluate swallowing function. Most longitudinal studies conducted swallowing evaluation at baseline and discharge; however, they did not focus on dynamic changes in swallowing function.

#### 3.4.2 Assessment methods of swallowing function evaluation in DoC patients

Almost all swallowing evaluation included in the study were performed by a clinician or physical therapist. We summarized the swallowing function assessment methods reported in the included studies into three categories: clinical assessment methods, scale-based assessment methods, and instrument-based assessment methods. These methods can be further subdivided into four, five, and three specific approaches, respectively. Overall, clinical assessment methods were the most commonly used across studies, followed by instrument-based methods and scale-based methods. Additionally, three studies employed a combination of clinical-based and instrument-based assessments (Mélotte et al., 2021; Peters et al., 2021; Bremare et al., 2016); and four studies adopted a combination of scale-based and instrument-based assessments (Prum et al., 2023; Moriyama et al., 2023; Jang et al., 2023; Herr et al., 2024).

##### 3.4.2.1 Clinical methods for swallowing function assessment

Clinical Evaluation methods for assessing swallowing function in patients with DoC include observation, the dye test, palpation, and the Kubota Drinking Test. These methods are least-invasive and bedside application, but vary in their complexity, with observation being the least invasive and easiest to apply, while Palpation offers a simple tactile assessment. The dye test and Kubota Drinking Test provide more specific diagnostic information, bridging the gap between observational and more invasive methods.

The observational method is the most frequently used clinical methods to assess swallowing function among included studies. The observation method entails researchers monitoring various indicators at the patient’s bedside, such as increased jaw muscle tension, lip gripping or tongue thrusting, throat secretions, saliva aspiration, cough reflex (number of coughs), movements of tongue and cheek muscles, number of swallows, aspiration of swallowed food, food residue in the mouth, changes in voice, and swallowing time parameters (Mélotte et al., 2021; Peters et al., 2021; Bremare et al., 2016; Wu et al., 2016; Wang et al., 2023). Among these, one study reported on the accuracy of the assessments for predicting consciousness alteration. Particularly, the sensitivity and specificity of the initiation of swallowing for DoC patients was 83.33% [95% CIs (36, 100%)] and 92.31% [95% CIs (64, 100%)], respectively (Peters et al., 2021).

One study utilized the dye test to assess swallowing function (Zhang N. et al., 2021). The dye test is a more invasive method that involves inflating the patient’s tracheostomy cuff to 30 cmH_2_O and adjusting subglottic suction to a negative pressure of 20–40 mmHg. Subsequently, 30 mL of dyed water (orange juice) is injected into the patient’s mouth using a syringe, followed by subglottic suction. This method assesses the presence of coughing, the number and timing of swallows during drinking, and is particularly useful for detecting subtle swallowing dysfunctions like silent aspiration that may not trigger a visible cough response (Zhang N. et al., 2021).

The palpation method requires researchers to gently place their fingers on the subject’s thyroid cartilage and hyoid bone, allowing the patient to swallow repeatedly to observe the number of swallows. Researchers feel the thyroid cartilage and hyoid bone pass over their fingers and return to the original position, indicating a complete swallow (Theytaz et al., 2023). This method offers a tactile means to assess the mechanics of swallowing and is relatively easy to perform at the bedside. However, this method require the individuals being assessed to have a certain level of swallowing reflex and cooperation. In one study, this method was used as the evaluation standard to estimate an invalidated technique for swallowing act assessment (Theytaz et al., 2023).

Among the included studies, three referred the Kubota Drinking Test to assess patients’ swallowing status (Wang et al., 2019; Zhang N. et al., 2021; Zhang et al., 2017). The Kubota Drinking Test involves administering 5–10 mL of water to the patient; if obvious coughing occurs, it is directly judged as abnormal. If no obvious coughing is observed, the patient is instructed to drink 30 mL of warm water “as usual,” and the process is observed and recorded (Chinese Rehabilitation Medicine Association Swallowing Disorder Rehabilitation Professional Committee, 2023). It is straightforward and commonly used in clinical practice for quick evaluation of swallowing status.

##### 3.4.2.2 Scale evaluation

Five scales are utilized to assess swallowing function in patients with DoC: the Functional Oral Intake Scale (FOIS) (Prum et al., 2023; Moriyama et al., 2023), the Penetration Aspiration Score (PAS) (Jang et al., 2023; Jakobsen et al., 2019), the Swallowing Assessment in Disorders of Consciousness (SWADOC) (Herr et al., 2024), the Standardized Swallowing Assessment (SSA) (Herr et al., 2024), and the Modified Mann Assessment of Swallowing Ability (MMASA) (Zhang C. H. et al., 2021). These scales vary in their focus, assessing aspects such as oral intake levels, risk of aspiration, and specific swallowing function in DoC patients. By summing the scores of the assessment items for degree classification, they provide a numerical and more standardized criteria to ensure consistency and reliability in clinical evaluation across different patient populations.

There are three studies using FOIS as the assessment tool for dysphagia among patients with DoC, which assesses the severity of dysphagia based on the patient’s prior oral intake activities (Prum et al., 2023; Moriyama et al., 2023; Jakobsen et al., 2019). It is divided into seven levels, with higher levels indicating better oral intake function. Levels 1 to 3 assess the degree of tube feeding dependence in patients unable to eat orally, while levels 4 to 7 assess the types and characteristics of oral intake in patients not requiring tube feeding. One study showed that the recovery of oral feeding in severely brain-injured patients was indicated as their levels of improved consciousness (Prum et al., 2023).

One study used PAS as the swallowing function assessment tool, which encompasses three dimensions related to swallowing safety: depth of airway invasion, response to airway invasion, and clearance of material from the airway (Jang et al., 2023). The scale is divided into eight levels, with level 1 indicating no food entry into the airway and level 8 indicating food reaching below the vocal cords and unable to be cleared (Crary et al., 2005). The highest score of airway penetration (contrast entering the laryngeal vestibule) and aspiration (contrast entering below the true vocal cords) during multiple swallows is taken as the final assessment score. A lower PAS score indicates better maintenance of swallowing ability in DoC patients, with appropriate responses to materials in the oropharyngeal cavity and reduced airway invasion (Jang et al., 2023).

SWADOC comprises both quantitative and qualitative components. The qualitative component involves therapists gathering detailed patient histories, observing consciousness levels, basic oral and facial conditions, saliva swallowing reflex, breathing, phonation, etc., and utilizing 5 mL of viscous solution for functional testing to evaluate lip and tongue function and aspiration during swallowing (Mélotte et al., 2021). The quantitative component primarily evaluates eight items: oral phase initiation, saliva secretion, lip and tongue function, pharyngeal reflex, airway protection, etc. The higher the score, the better the swallowing ability of DoC patients is preserved. The Herr’s et al. (2024) study evaluated the clinical application of SWADOC, which demonstrated good internal consistency and test-retest reliability.

SSA was used in one included study (Herr et al., 2024), which consists of three parts: initially, observing the patient’s consciousness, breathing, pharyngeal reflex, and spontaneous coughing, with a total score of 8 to 23. After scoring these basic conditions, the patient is instructed to swallow 5 mL of water, with swallowing and laryngeal function observed, scoring 5 to 22; if no abnormalities are detected in the first and second assessments, the patient is instructed to swallow 60 mL of water, observing the time required for swallowing and the presence of coughing, scoring 5 to 12. The lower the total score, the better the swallowing function (Ellul et al., 1997). This assessment requires the patient to cooperate in the drinking trial.

One study referred the MMASA as the assessment tool (Zhang C. H. et al., 2021), which evaluates swallowing function through 12 items, including dysarthria, tongue muscle movement, pharyngeal reflex, cough reflex, and soft palate function. The total score is 100, with higher scores indicating better swallowing function.

##### 3.4.2.3 Instrument evaluation

Instrumental evaluation methods for assessing swallowing function in patients with DoC include Fiberoptic Endoscopic Evaluation of Swallowing (FEES) (Mélotte et al., 2021; Bremare et al., 2016; Prum et al., 2023), Video Fluoroscopy Swallowing Study (VFSS) (Peters et al., 2021; Prum et al., 2023; Moriyama et al., 2023), the Nox-T3 sleep monitoring device (Theytaz et al., 2023), and Surface Electromyography (SEMG) (Jakobsen et al., 2019; Theytaz et al., 2023). These methods differ in their application, technical requirements, and the type of data they provide. However, they share the common goal of assessing the safety and efficiency of swallowing in DoC patients, each contributing specific advantages and limitations to the clinical evaluation.

FEES provides real-time and direct visualization of the pharyngeal phase of swallowing, involving the use of a flexible endoscope that insert through the nasal passage to access the oropharynx and hypopharynx, allowing observation of structures such as the epiglottis, vallecula, tongue base, pharyngeal wall, larynx, and pyriform sinuses. It assesses their movements during breathing, phonation, coughing, breath-holding, and swallowing foods of varying consistencies (Chinese Rehabilitation Medicine Association Swallowing Disorder Rehabilitation Professional Committee, 2023). However, it may be limited by the patient’s ability to tolerate the procedure. Bremare et al. (2016) showed that among their 11 included patients, only five patients were able to execute a movement on request. Besides, they were unable to reach a conclusion for three of them.

VFSS is an another widely used instrumental assessment in patients with DoC who exhibit swallowing difficulties, and is less invasive than the FEES method. In Jang’s et al. (2023) study, patients’ performance on videofluoroscopic swallowing study could well predict the recovery of impaired consciousness. This method provides dynamic, X-ray-based visualization of the entire swallowing process from mouth to esophagus, involving the swallowing movements of the mouth, pharynx, larynx, and esophagus using X-ray fluoroscopy. This procedure requires the patient to ingest food or liquid mixed with a contrast medium (barium). Physicians employ lateral and anteroposterior imaging to observe patients swallowing contrast agents of varying viscosities and doses, thereby evaluating abnormalities in the oral preparatory, oral, pharyngeal, and esophageal phases (Chinese Rehabilitation Medicine Association Swallowing Disorder Rehabilitation Professional Committee, 2023). VFSS is able to visualize the entire swallowing process. However, it involves radiation exposure, which limits its frequency of use and may be a concern in certain patient populations.

SEMG provides non-invasive monitoring of muscle activity involved in swallowing, which directly assesses the neuromuscular function of the oropharynx by recording the total electrical activity within the electrode range (Jakobsen et al., 2019; Theytaz et al., 2023). It can also measure maximum laryngeal height, laryngeal elevation speed, swallowing frequency, and hyoid-laryngeal complex activity during swallowing (Alcan and Zinnuroğlu, 2023). SEMG allows continuous monitoring of muscle function, but it may not provide direct information about the airway or the pharyngeal phase of swallowing.

The Nox-T3 sleep monitoring device is used to record physiological signals during sleep through both built-in and patient-attached sensors. It provide data on blood oxygen levels, thoracoabdominal respiratory movements, positional changes, and nasal airflow pressure signals. While it is effective for detecting sleep-related swallowing issues such as aspiration during sleep, it does not directly evaluate the mechanics of swallowing. Its accuracy depends on the correct placement of sensors and the quality of the sleep study. Surface electromyography from the submental and peripharyngeal areas, connected to the Nox-T3 sleep monitoring device, was used to capture swallowing actions in 14 DoC patients. The Nox-T3 sleep monitoring device method demonstrates a sensitivity of 95% and a specificity of 99% in identifying swallowing events (Theytaz et al., 2023).

### 3.5 Intervention for swallowing function in patients with DoC

#### 3.5.1 Timing of swallowing function intervention in DoC patients

The included study encompasses several swallowing intervention methods, including sensory stimulation therapy (SST), K-point stimulation, neuromuscular electrical stimulation therapy, facial oral tract therapy (FOTT), intermittent ora-esophageal tube feeding (IOE), and acupuncture. These interventions often lacked a clearly defined initiation period for implementation, such as the specific duration after a change in consciousness before they were applied. Most interventions typically lasts for a period of 3 to 4 weeks, with sessions held regularly during this time.

#### 3.5.2 Methods of swallowing function intervention in DoC patients

Of the included studies, only two involved nurses as implementers of swallowing interventions (Zhang C. H. et al., 2021; Zhang et al., 2023). Five studies employed SST for swallowing function training (Wu et al., 2016; Zhang et al., 2017; Zhang C. H. et al., 2021; Zhang et al., 2023; Wang et al., 2023). SST is most appropriate for patients with mild to moderate DoC, where some level of sensory responsiveness remains. This therapy involves the use of frozen cotton swabs or swabs soaked in chili juice to massage and stimulate the soft palate, palatal arch, tongue base, and posterior pharyngeal wall. During the intervention, both cold and chili juice stimulations can enhance swallowing actions and cheek muscle movements, with chili juice stimulation proving more effective than cold stimulation (Wu et al., 2016). However, the effectiveness of SST varies widely depending on the patient’s level of consciousness and responsiveness, with minimal benefit in deeply unconscious or minimally responsive patients.

Two studies utilized K-point stimulation to enhance swallowing function training in DoC patients (Zhang et al., 2017; Zhang et al., 2023). The K-point is situated in the retromolar triangle, specifically at the central position of the pterygomandibular raphe and palatoglossal arch. Stimulating the K-point can enhance nerve ending sensitivity, improve sensory nerve impulse transmission, induce tongue movement, and promote the swallowing reflex (Kojima et al., 2002). This method is non-invasive and has shown potential in enhancing motor responses, improving the coordination of the swallowing process. It can also facilitate the restoration of autonomic function. Building on the aforementioned temperature and pain stimulation therapies, Zhang et al. (2017) utilized an ice-acid swab to moderately stimulate the K-point, further enhancing swallowing function in DoC patients, particularly during the oral phase. Additionally, some studies employed cotton swabs soaked in essential balm for K-point stimulation. Essential balm possesses a distinctive odor that strongly stimulates taste and smell, and K-point stimulation using essential balm is more effective in improving swallowing function than conventional ice-acid swab K-point stimulation (Zhang et al., 2023).

Three studies employed neuromuscular electrical stimulation to enhance swallowing function in patients with DoC (Zhang et al., 2017; Zhang et al., 2023; Wang et al., 2023), which showed an improvement of consciousness state and oral function among patients with DoC. This therapy involves the use of an electrical stimulator, with electrodes positioned vertically along the midline: the first electrode is placed above the hyoid bone, the second near the first above the thyroid cartilage notch, and the third and fourth electrodes above the cricoid cartilage, adjacent to the second electrode. Compared to sensory stimulation therapy, neuromuscular electrical stimulation resulted in improved SSA scores, Kubota Drinking Test scores, and increased activity of the hyoid-laryngeal complex in DoC patients (Wang et al., 2023). Nevertheless, it is invasive in that it requires the use of electrodes placed on the skin, and patients may experience discomfort.

One study utilized FOTT for swallowing function training in patients with DoC. FOTT involves therapists using common objects and functional activities from daily life to address four areas: swallowing, oral hygiene, breathing/phonation, and speech communication. It employs tactile stimulation to activate sensory inputs or muscle movements as substitutes for verbal instructions in training patients unable to perform executive actions (Mortensen et al., 2016). Jakobsen et al. (2019) enhanced non-verbal swallowing action training for DoC patients by building upon FOTT training. Although both groups showed increased scores on the PAS and FIOS, the intervention group demonstrated a higher swallowing frequency than the control group. The therapy requires active participation from the patient, which may be challenging in patients with more severe DoC.

Zhang C. H. et al. (2021) used an IOE method to preserve the swallowing function among patients with chronic unconsciousness. IOE is a novel nutritional method involving the insertion of a feeding tube through the mouth into the stomach or esophagus before meals, allowing nutrient-rich liquid food, water, and medication to be injected into the stomach, with the tube removed immediately after injection. IOE is most appropriate for patients with severe DoC who cannot swallow safely or effectively and are at risk for aspiration. Using IOE, DoC patients not only increased their MMASA scores but also improved their consciousness and nutritional status (Zhang C. H. et al., 2021).

**Figure 3:**
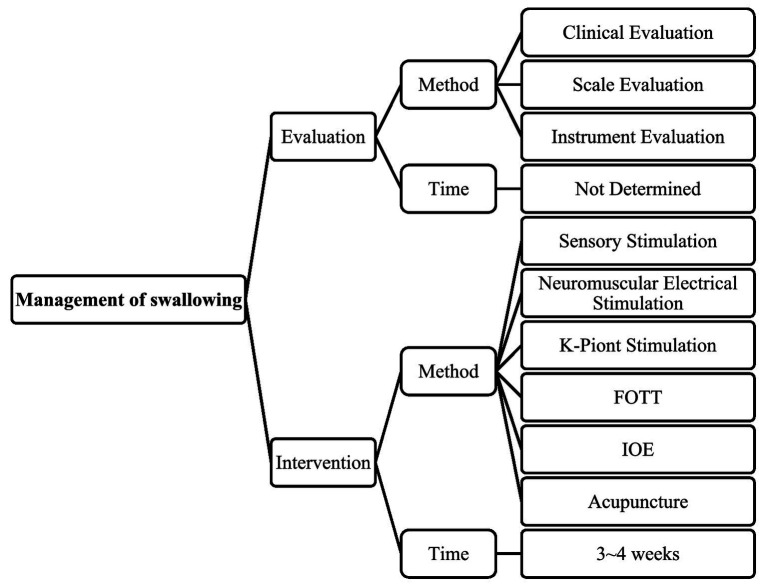
Management of swallowing function in patients with DoC. FOTT, facial oral tract therapy; IOE, intermittent ora-esophageal tube feeding.

In Wang’s et al. (2023) study, traditional Chinese therapy, acupuncture therapy, was utilized. Acupuncture therapy involves rehabilitation therapists selecting specific acupoints such as Lianquan, Jinjin, Yuye, Jia Lianquan, Yifeng, and Fengchi. A 40.0 mm × 0.3 mm needle is inserted obliquely to a depth of 0.5 to 0.8 cun, with lifting, thrusting, and twisting techniques performed once qi is obtained, aiming for the patient to feel soreness, numbness, distension, or to vocalize. Combining acupuncture therapy with sensory and neuromuscular electrical stimulation treatments can further improve swallowing function in DoC patients, enhance tongue muscle movement, shorten swallowing time, and increase hyoid-laryngeal complex activity (Wang et al., 2023). Acupuncture shares similarities with K-point stimulation in that both involve the stimulation of specific body points to enhance physiological function, yet acupuncture focuses on broader systemic effects rather than being specific to swallowing. In summary, the management of swallowing function in DoC patients is depicted in Figure 3.

## 4 Discussion

The study identified inconsistency in the timing and assessment method of swallowing evaluation for patients with DoC. The assessment modalities encompassed clinical evaluation, scale-based assessment, and instrumental techniques across the reviewed studies. Swallowing rehabilitation interventions, as reviewed, included sensory stimulation, neuromuscular electrical stimulation, and acupuncture, albeit with generally low methodological quality observed among the studies.

Early initiation of swallowing evaluations proves crucial in promptly identifying dysphagia to mitigate risks such as aspiration pneumonia, malnutrition, and dehydration. The reported prevalence of swallowing disorders among studies varies significantly across studies, with estimates ranging from 30 to 100%. This discrepancy may stem from heterogeneity in study populations. For example, some studies include populations in a MCS (Yang et al., 2017; Wang et al., 2019; Prum et al., 2023; Jang et al., 2023; Herr et al., 2024), characterized by the presence of conscious behavior, while others focus on comatose populations (Peters et al., 2021; Bremare et al., 2016; Moriyama et al., 2023), in which there is a complete absence of self-awareness and conditioned reflexes. Additionally, there are methodological differences such as the use of screening tools (e.g., bedside clinical exams) versus instrumental assessments (e.g., FEES). This may lead to discrepancies in reported prevalence due to differences in assessment accuracy and identification capabilities. Regular assessments are essential for dynamic monitoring of swallowing function and timely intervention, responsive to neurological and physical changes in patients. Among the included studies, initial assessments often occurring significantly later after the onset of the condition. Despite guideline recommendations advocating for immediate screening of suspected dysphagia (Chinese Rehabilitation Medicine Association Swallowing Disorder Rehabilitation Professional Committee, 2023), a gap persists between clinical practice and guideline adherence, possibly due to ambiguous tool selection guidance for DoC patients. This results in healthcare professionals being unable to select appropriate assessment tools in specific clinical contexts and missing patient recovery opportunity. Therefore, it is imperative to explore evidence-based methods and tools for assessing swallowing function in DoC patients.

The reviewed literature indicates that the assessment and rehabilitation of swallowing function in patients with DoC are primarily conducted by rehabilitation therapists, with only three studies involving nurse participation (Zhang N. et al., 2021; Zhang C. H. et al., 2021; Zhang et al., 2023). Not all hospitals have trained professionals available for continuous assessments, nor do they possess the necessary equipment. Nurses, who have the most frequent contact with patients, are often the first to assess swallowing ability. Therefore, nurses play a critical role in swallowing assessments, particularly in bedside evaluations like the KDT (Martino et al., 2005). This aligns with evidence from nursing literature (Rose et al., 2025; Jones and Porterfield, 2020), where trained nurses effectively implement standardized screening tools, contributing to early identification of dysphagia and reducing aspiration risks in vulnerable populations. However, a qualitative study on nurses’ screening for swallowing difficulties in acute stroke patients suggests that nurses are inadequately prepared to manage such difficulties and face challenges during initial assessments (de Jesus Oliveira et al., 2020). Therefore, providing nurses with training in swallowing assessment offers long-term and ongoing benefits for managing swallowing function in DoC patients.

Clinical, scale-based, and instrumental assessments are viable approaches for evaluating swallowing function in DoC patients, although a standardized dysphagia screening protocol remains absent. Clinical assessments allow for repeatable evaluations with high specificity but low sensitivity (Sherman et al., 2018). Methods like observation and palpation are influenced by subjective factors and varying times, which can affect judgments. The dye test, aimed at tracheostomized patients, relies on the Kubota Drinking Test, which uses clinical symptoms and subjective feelings to assess swallowing function. However, it has a high missed diagnosis rate for aspiration in those with abnormal pharyngeal reflexes, making it unreliable. These clinical assessments have low sensitivity and specificity, and further research is needed to confirm their safety and acurity in assessing swallowing function in DoC patients (Zhang et al., 2020).

Scale assessments, such as FOIS and PAS, present non-invasive bedside monitoring options, each with distinct strengths and limitations in assessing swallowing safety. FOIS is not constrained by the patient’s level of consciousness and can effectively assess the safety of oral feeding in DoC patients, indirectly evaluating their swallowing function (Mortensen et al., 2020). PAS used as an interpretive tool for FEES and VFSS results, demonstrates a high positive correlation and consistency with both (Yoon et al., 2019). The scoring of the PAS varies across statistical methods and research practices. Abnormalities or changes in the patient’s anatomical structures, the evaluator’s confidence, and the accuracy of reference standards can all impact the precise use of the PAS (Kitila et al., 2024). SSA is suitable for screening swallowing disorders in patients with neurological dysfunction, but it requires patient consciousness and cooperation (Boaden et al., 2021). In patients with severe traumatic brain injury, the SSA showed reduced likelihood of identifying swallowing difficulties as positive (Bérubé et al., 2024). The Chinese expert consensus on neurocritical care rehabilitation recommends MMASA for assessing swallowing function in neurocritical patients. The MMASA exhibits high sensitivity and specificity in predicting swallowing difficulties (Ni et al., 2018). The SWADOC scale specifically evaluates the swallowing characteristics of DoC patients, which is available in French and English, demonstrating good reliability and validity in multicenter prospective cohort studies in France and Belgium (Herr et al., 2024). Future research should focus on localizing and validating the SWADOC scale for use in more regions and countries. Additionally, we recommend studies conducting multi-time-point measurements of swallowing function so that address the longitudinal trends in changes of swallowing function.

Instrumental assessments like FEES and VFSS, are considered gold standards (Labeit et al., 2022). Theoretically, all patients with swallowing disorders should undergo FEES or VFSS evaluations. FEES serves as the best alternative to VFSS, enabling safe and economical bedside assessments, particularly suitable for ICU patients (Chen et al., 2023). However, systematic reviews suggest insufficient evidence supporting the use of FEES for assessing swallowing function in patients with DoC (Checklin et al., 2022). Additionally, conducting a FEES assessment prior to oral feeding in patients is deemed unnecessary in some studies (Kjaersgaard et al., 2014). Emerging technologies like the Nox-T3 sleep monitor and electromyography show promise in providing convenient and accurate data for assessing swallowing function in DoC patients. The Nox-T3 avoids patient discomfort and radiation exposure risks, making it potentially suitable for long-term monitoring of patients with consciousness disorders and other functions, such as sleep apnea (Xu et al., 2017). However, the existing study on DoC patients is a small-scale, single-center trial. Besides, cost-effectiveness analyses are absent considering the device’s high upfront costs may limit accessibility in resource-constrained settings (Alakuijala and Salmi, 2016). There is still an urgent need to develop nursing techniques and equipment tailored to the unique physiological characteristics DoC patients.

This study included seven interventional studies on the rehabilitation of swallowing function in DoC patients, comprising five domestic studies and one international study. Among these, two were randomized controlled trials, but neither reported methods of random allocation or blinding, indicating a generally low quality of interventional research. Therefore, the effectiveness of sensory stimulation, neuromuscular electrical stimulation, K-point stimulation FOTT, IOE, and acupuncture in the intervention of swallowing function in DoC patients lacks solid evidence from evidence-based medicine. Currently, no standardized protocol exists for rehabilitating swallowing function in this patient population. Several factors contribute to this situation. First, there is a lack of DoC patients meeting the inclusion criteria in clinical settings, and patients are prone to dropout during interventions. Secondly, the tools for assessing swallowing in DoC patients are not yet fully developed, and research on swallowing mechanisms and influencing factors is insufficient. Thirdly, organizational challenges necessitate specialized resources for effective swallowing rehabilitation, which may not always be available outside regular working hours. Lastly, only one included study mentioned that intervention implementers involved family members in the swallowing function rehabilitation program (Zhang C. H. et al., 2021). Research has found that caregiver involvement in patient rehabilitation can significantly improve the patient’s depression and anxiety, as well as greatly reduce the caregiver’s guilt (Mori et al., 2024). Therefore, to support caregivers, healthcare professionals should explore and integrate new multidisciplinary approaches into dysphagia rehabilitation strategies, developing personalized rehabilitation care plans to enhance patient functional recovery and quality of life. This will also promote the integration of nursing with multidisciplinary, multi-field, and multi-professional development, helping nurses identify and solve problems from multiple perspectives and focus on global nursing frontier issues.

## 5 Limitations

The study systematically examined swallowing assessments and rehabilitation techniques for DoC patients, identifying existing gaps and challenges. However, there are also some limitations that need to be considered: firstly, a key limitation of this review lies in the marked heterogeneity across studies regarding the dimensions of consciousness states, etiological profiles, and sample sizes. Such etiological and phenotypical divergence likely inflated results heterogeneity, reducing the certainty of pooled estimates, which should be interpreted with caution. Additionally, most reviewed studies had methodological weaknesses, limiting the reliability of findings. Furthermore, the absence of validated and widely accepted screening tools and protocols for DoC patients restricts the generalizability of recommendations.

## 6 Conclusion

In summary, the methods for assessing swallowing function in patients with DoC mainly include clinical assessment, scale assessment, and instrumental assessment. Swallowing function rehabilitation interventions include sensory stimulation, K-point stimulation, FOTT, IOE, neuromuscular electrical stimulation, and acupuncture therapy. However, there is still a lack of standardized methods for assessing and intervening in the swallowing function of patients with consciousness disorders. Although different assessment methods have their limitations, we recommend that in resource-limited settings, simple clinical assessments be used for initial screening. Additionally, we suggest further validation of different scales’ psychological reliability across regions and populations (such as SWADOC). Studies could also integrate economic evaluations to assess the cost-effectiveness of these tools, especially instrumental assessment tools. Combining various assessment methods may enhance the ability to evaluate swallowing disorders in DOC patients. Our finding not only provides a new perspective on understanding the existing assessment methods of swallowing disorders in these patients, but also offers potential strategies for developing new approaches and bringing new therapeutic hope to patients. Meanwhile, we recommend conducting high-quality randomized controlled trials in the future to explore the effectiveness of assessment and intervention plans.

## Data Availability

The datasets presented in this study can be found in online repositories. The names of the repository/repositories and accession number(s) can be found in the article/Supplementary material.

## Footnotes

^1^ https://doi.org/10.17605/OSF.IO/SURBY
