# Correction: Swallowing function management in patients with disorders of consciousness: a scoping review

**DOI:** 10.3389/fnins.2025.1665746

**Published:** 2025-07-24

**Authors:** Hua Long, Bixia Lu, Qiao Tan, Dongmei Dai, Fengfei Zeng

**Affiliations:** ^1^Department of Clinical Nursing, Second Xiangya Hospital, Central South University, Changsha, China; ^2^Department of Neurosurgery, Second Xiangya Hospital, Central South University, Changsha, China; ^3^Xiangya Boai Rehabilitation Hospital, Changsha, China

**Keywords:** disorders of consciousness, swallowing disorder, scoping review, assessment, intervention

An incorrect number was provided for [Grant No. KYZ202400994]. The correct number is [Grant No. 2024JJ9118].

The original version of this article has been updated.

