# Supplementary material for: Swallowing function management in patients with disorders of consciousness: a scoping review
Source: Front Neurosci. 2025 Apr 16;19:1595393. doi: 10.3389/fnins.2025.1595393 (PMC12040949; doi:10.3389/fnins.2025.1595393)
Supplement: Supplementary file 1 [file Data_Sheet_1.pdf]

Searching terms for databases.

- **PubMed**

(("Consciousness Disorders"[Mesh] OR "Persistent Vegetative State"[Mesh] OR "Coma"[Mesh] OR "Unconsciousness"[Mesh] OR "Minimally Conscious State"[Mesh]) OR ("disorders of consciousness"[Title/Abstract] OR "vegetative state"[Title/Abstract] OR "unresponsive wakefulness syndrome"[Title/Abstract] OR "MCS"[Title/Abstract] OR "post-comatose state"[Title/Abstract] OR "prolonged coma"[Title/Abstract] OR (("traumatic brain injury"[Title/Abstract] OR "stroke"[Title/Abstract]) AND ("consciousness"[Title/Abstract] OR "awareness"[Title/Abstract]))))

AND

(("Deglutition Disorders"[Mesh] OR "Dysphagia"[Mesh] OR "Oropharyngeal Dysphagia"[Mesh] OR "Esophageal Dysphagia"[Mesh]) OR ("swallow* disorder*"[Title/Abstract] OR "swallow* dysfunction*"[Title/Abstract] OR "impaired swallow*"[Title/Abstract] OR "dysphagia"[Title/Abstract] OR "feeding difficult*"[Title/Abstract]))

- **Web of Science**

(TS=(Disorders of consciousness OR Persistent Vegetative State OR Coma OR Stupor OR Consciousness Disorder OR Disorder of Consciousness OR Disorders of Consciousness OR Consciousness, Level Depressed OR Depressed Level of Consciousness OR Minimally consciousstate OR Persistent Unawareness State OR Persistent Unawareness States OR State, Persistent Unawareness OR States, Persistent Unawareness OR Unawareness State, Persistent OR Unawareness States, Persistent OR PVS OR Persistent Vegetative State OR PVSs OR Persistent Vegetative State OR Vegetative State, Persistent OR Persistent Vegetative States OR State, Persistent Vegetative OR States, Persistent Vegetative OR Vegetative States, Persistent OR Vegetative State OR States, Vegetative OR State, Vegetative OR Vegetative States OR Minimally Conscious State OR Minimally Conscious States OR State, Minimally Conscious OR States, Minimally Conscious OR Permanent Vegetative State OR

Permanent Vegetative States OR State, Permanent Vegetative OR States, Permanent Vegetative OR Vegetative State, Permanent OR Vegetative States, Permanent OR Post-Traumatic Vegetative State OR Post Traumatic Vegetative State OR Post-Traumatic Vegetative States OR State, Post-Traumatic Vegetative OR States, Post-Traumatic Vegetative OR Vegetative State, Post-Traumatic OR Vegetative States, Post-Traumatic OR Post-Traumatic Unawareness State OR Post Traumatic Unawareness State OR Post-Traumatic Unawareness States OR State, Post-Traumatic Unawareness OR States, Post-Traumatic Unawareness OR Unawareness State, Post-Traumatic OR Unawareness States, Post-Traumatic OR Posttraumatic Unawareness State OR Posttraumatic Unawareness States OR State, Posttraumatic Unawareness OR States, Posttraumatic Unawareness OR Unawareness State, Posttraumatic OR Unawareness States, Posttraumatic OR Post-Comatose Unawareness State OR Post Comatose Unawareness State OR Post-Comatose Unawareness States OR State, Post-Comatose Unawareness OR States, Post-Comatose Unawareness OR Unawareness State, Post-Comatose OR Unawareness States, Post-Comatose OR Postcomatose Unawareness State OR Postcomatose Unawareness States OR State, Postcomatose Unaware)) AND TS=(Deglutition Disorders OR Deglutition Disorder OR Disorders, Deglutition OR Dysphagia OR Swallowing Disorders OR Swallowing Disorder OR Oropharyngeal Dysphagia OR Dysphagia, Oropharyngeal OR Esophageal Dysphagia OR Dysphagia, Esophageal OR Deglutition dysfunction OR Deglutition impairment OR Swallowing dysfunction OR Impaired swallowing OR Acataposis OR Swallow problem)

- **Embase**

1.exp consciousness disorder/ or exp vegetative state/ or exp coma/ or exp minimally conscious state/ (disorders of consciousness or persistent vegetative state or coma or stupor or unresponsive wakefulness syndrome or MCS or post-comatose state).ti,ab,kw.

2. exp deglutition disorder/ or exp dysphagia/ or exp oropharyngeal dysphagia/ or exp esophageal dysphagia/ (deglutition disorder* or dysphagia or swallowing disorder* or swallowing dysfunction* or impaired swallowing).ti,ab,kw.

3. 1 and 2

- **Clinical Trials**

(("disorders of consciousness" OR "vegetative state" OR "coma") AND ("swallowing disorder" OR "dysphagia"))

Study Type: Interventional Studies | Observational Studies

- **Cochrane**

#1 [mh "Consciousness Disorders"] OR [mh "Persistent Vegetative State"] OR [mh "Coma"] OR [mh "Minimally Conscious State"]

#2 (disorders of consciousness OR vegetative state OR coma OR MCS):ti,ab,kw

#3 #1 OR #2

#4 [mh "Deglutition Disorders"] OR [mh "Dysphagia"] OR [mh "Oropharyngeal Dysphagia"]

#5 (swallow* disorder* OR dysphagia OR deglutition disorder):ti,ab,kw

#6 #4 OR #5

#7 #3 AND #6

- **Scopus**

TITLE-ABS-KEY(("disorders of consciousness" OR "vegetative state" OR "coma" OR "minimally conscious state") AND ("swallowing disorder" OR "dysphagia" OR "deglutition disorder"))

- **Medline**

(("Consciousness Disorders"[Mesh] OR "Persistent Vegetative State"[Mesh] OR "Coma"[Mesh] OR "Unconsciousness"[Mesh] OR "Minimally Conscious State"[Mesh]) OR ("disorders of consciousness"[Title/Abstract] OR "vegetative state"[Title/Abstract] OR "unresponsive wakefulness syndrome"[Title/Abstract] OR "MCS"[Title/Abstract] OR "post-comatose state"[Title/Abstract] OR "prolonged coma"[Title/Abstract] OR (("traumatic brain injury"[Title/Abstract] OR "stroke"[Title/Abstract]) AND ("consciousness"[Title/Abstract] OR "awareness"[Title/Abstract]))))

AND (("Deglutition Disorders"[Mesh] OR "Dysphagia"[Mesh] OR "Oropharyngeal Dysphagia"[Mesh] OR "Esophageal Dysphagia"[Mesh]) OR ("swallow*

disorder*"[Title/Abstract] OR "swallow* dysfunction*"[Title/Abstract] OR "impaired swallow*"[Title/Abstract] OR "dysphagia"[Title/Abstract] OR "feeding difficult*"[Title/Abstract]))

- **CNKI**

摘要=('意识障碍' + '植物状态' + '昏迷') AND 摘要=('吞咽障碍' + '吞咽困难' + '吞咽康复')

- **WangFan**

主题:("意识障碍" OR "持续性植物状态" OR "昏迷") AND 主题:("吞咽功能" OR "吞咽障碍" OR "吞咽康复")

- **Vip**

K=("意识障碍" | "植物人" | "昏迷") * K=("吞咽障碍" | "吞咽困难" | "吞咽康复")

- **Sinomed**

#1 主题词: 意识障碍/全部树/ OR 植物人/全部树/ OR 昏迷/全部树/

#2 自由词: "意识障碍" OR "植物状态" OR "微意识状态"

#3 #1 OR #2

#4 主题词: 吞咽障碍/全部树/ OR 吞咽困难/全部树/

#5 自由词: "吞咽功能" OR "误吸风险"

#6 #4 OR #5

#7 #3 AND #6
